# Short-term ocular dominance plasticity is not modulated by visual cortex tDCS but increases with length of monocular deprivation

**DOI:** 10.1038/s41598-023-33823-7

**Published:** 2023-04-24

**Authors:** Xiaoxin Chen, William Bobier, Benjamin Thompson

**Affiliations:** 1 School of Optometry & Vision Science, University of Waterloo, Waterloo, ON Canada; 2 Centre for Eye and Vision Research, 17W Science Park, Hong Kong, China; 3 Liggins Institute, University of Auckland, Auckland, New Zealand

**Keywords:** Sensory processing, Visual system

## Abstract

Transcranial direct current stimulation (tDCS) of the occipital lobe may modulate visual cortex neuroplasticity. We assessed the acute effect of visual cortex anodal (a-)tDCS on ocular dominance plasticity induced by short-term monocular deprivation (MD), a well-established technique for inducing homeostatic plasticity in the visual system. In Experiment 1, active or sham visual cortex tDCS was applied during the last 20 min of 2-h MD following a within-subjects design (n = 17). Ocular dominance was measured using two computerized tests. The magnitude of ocular dominance plasticity was unaffected by a-tDCS. In Experiment 2 (n = 9), we investigated whether a ceiling effect of MD was masking the effect of active tDCS. We replicated Experiment 1 but used only 30 min of MD. The magnitude of ocular dominance plasticity was decreased with the shorter intervention, but there was still no effect of active a-tDCS. Within the constraints of our experimental design and a-tDCS parameters, visual cortex a-tDCS did not modulate the homeostatic mechanisms that drive ocular dominance plasticity in participants with normal binocular vision.

## Introduction

Transcranial direct current stimulation (tDCS) involves the delivery of a weak direct electrical current to targeted cortical sites via electrodes placed on the scalp. tDCS modulates neural excitability of the stimulated brain area in a polarity-dependent manner^1,2^. In the motor cortex, anodal tDCS (a-tDCS) elevates motor evoked potential (MEP) amplitude, indicating increased cortical excitability, whereas cathodal tDCS (c-tDCS) has the opposite effect^3^. tDCS may alter neural membrane potentials and increase or decrease the activity of sodium and calcium channels, therefore altering the probability of action potentials^4,5^. In addition, a-tDCS may modulate neurotransmission by facilitating serotonin, dopamine and glutamate signaling and attenuating the inhibitory GABAergic system, thus producing after effects on neural activity that outlast the stimulation itself^5–9^.

When applied to the visual cortex, a-tDCS has effects that are consistent with reduced cortical inhibition. For instance, visual cortex a-tDCS enhanced visually evoked potential (VEP) amplitudes in adults with normal vision for up to 50 min post stimulation, indicating increased cortical excitability, perhaps due to reduced inhibition^10^. Visual cortex a-tDCS also improves vernier acuity, Snellen acuity, contrast sensitivity for high spatial frequencies^11^, crowding in peripheral vision^12^ and can augment the effect of visual perceptual learning (VPL)^13^. In addition, a-tDCS effects have been examined among patients with amblyopia, a neurodevelopmental vision disorder characterised by chronic suppression of one eye. Spiegel et al. observed that visual cortex a-tDCS improved contrast sensitivity in some adults with amblyopia^14^ and enhanced the effect of videogame-based dichoptic therapy on stereoacuity^15^. Ding et al.^16^ demonstrated that a-tDCS increased VEP amplitude and improved contrast sensitivity in both adults with normal vision and adults with amblyopia. These studies indicate that a-tDCS can modulate visual cortex function and enhance visual cortex neuroplasticity.

To further examine the short-term effect of a-tDCS on visual cortex plasticity, we tested the hypothesis that a single session of visual cortex a-tDCS would enhance ocular dominance plasticity induced by short term monocular deprivation (MD). This is a well-established paradigm for producing homeostatic neuroplasticity within the human binocular visual system that causes a transient increase in deprived eye dominance^17–20^. Ocular dominance plasticity involves mechanisms that may be modulated by a-tDCS. These include reduced GABAergic inhibition within the visual cortex and a transient increase in contrast gain for the deprived eye^18,20,21^.

In our first experiment, participants received MD for 2 hours and anodal, cathodal and or sham tDCS was applied to the visual cortex during the final 20 min of MD. We assessed whether ocular dominance changes were augmented by a-tDCS. Cathodal tDCS (c-tDCS) was included as an active control condition. In Experiment 2, we reduced the deprivation time to 30 min to test for ceiling effects in the magnitude of ocular dominance plasticity. We found no effects of a-tDCS. However, we did observe weaker ocular dominance plasticity following 30 min compared to 120 min of monocular deprivation.

## Methods

### Participants

All participants had normal vision as defined by aided visual acuity of at least 20/20 in each eye and stereoacuity of at least 40 s of arc. Visual acuity was measured by an ETDRS chart (Precision Vision, USA). Stereoacuity was measured by the Titmus circle test (Stereo Optical Company, Inc., USA). We excluded participants who were unable to fuse dichoptic images reliably or had an ocular dominance > 0.7 (one eye significantly more dominant than the other) as measured by either of our ocular dominance tests described below. Additionally, in line with guidelines in the tDCS literature, we excluded participants with a history or immediate family history of epilepsy or seizures, an implanted medication pump, a pacemaker, a defibrillator, metal implants in the head, heart disease, skin conditions at the electrode sites, pregnancy, hearing loss, recurring headaches, head injury, psychiatric conditions or psychoactive medication. Participants were instructed to avoid alcohol (more than one standard drink per hour) within 24 h of testing, avoid caffeinated beverages within 3 h of testing and ensure at least 5 h of sleep before each visit. All participants provided written informed consent prior to participation. This study was reviewed and approved by the University of Waterloo Research Ethics Board and adhered to the Declaration of Helsinki.

### Visual stimuli for ocular dominance measurement

**Figure 1:**
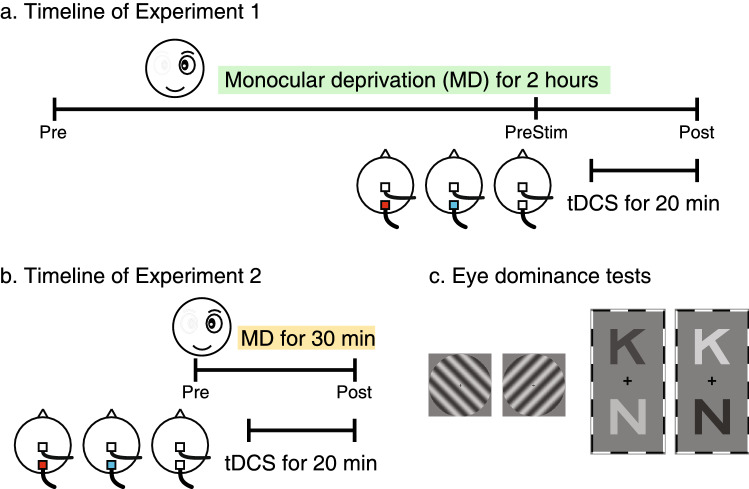
(**a**) Illustration of the timeline in Experiment 1. Ocular dominance was measured at baseline (Pre), before tDCS at the 90th minute of MD (PreStim) and immediately after 2 h of MD (Post). (**b**) Illustration of the timeline for Experiment 2. Ocular dominance was measured at baseline (Pre) and immediately after 30 min of MD (Post). In both experiments, a-tDCS, c-tDCS or sham stimulation was delivered to the visual cortex during the final 20 min of MD. (**c**) Examples of the two ocular dominance tests. Participants viewed the stimuli dichoptically. In the grating rivalry test, participants continuously pressed one of four buttons to indicate their perception. In the letter-polarity test, participants pressed the up or down arrow key to indicate the letter that they perceived as brighter than the other.

We employed two measures of ocular dominance, a grating rivalry test and a letter-polarity test (Fig. 1). For both tests, visual stimuli were presented on a Windows computer (Lenovo M710s, Intel i7-7700, 8 GB RAM) with an Asus VG279 monitor (60 Hz refresh rate, 1920 × 1080 resolution). The grating rivalry test stimuli were generated using MATLAB R2018a (Mathworks Inc., USA) with Psychtoolbox 3.0.18 extensions. The letter-polarity test stimuli were prepared using the Psychopy module for Python 3.6.6. Stimuli were dichoptically presented through a mirror stereoscope. Participants rested their head on a chinrest and viewed the stimuli at a distance of 86 cm.

### Ocular dominance tests

The two ocular dominance tests have been described in detail in a previous paper^22^. Briefly, in the grating rivalry test (Fig. 1), participants viewed two stationary circular gratings (2° diameter, 2 cpd, orthogonally oriented, + 45° and − 45°), one in each eye. They were instructed to continuously indicate their binocular rivalry perception using one of four keys on a keyboard across six, one-minute trials. The duration of each rivalry percept (deprived eye dominance, non-deprived eye dominance, superimposition, and piecemeal) was measured. Trials with less than 50 s of button press responses were excluded from the analysis. As a result, 9 trials were removed from a total of 612 trials in Experiment 1; none were removed in Experiment 2. Average exclusive (deprived eye and non-deprived eye) and mixed (superimposition and piecemeal) durations were calculated. Ocular dominance was calculated as OD_rivalry_ = $\frac{{{\text{d}}_{{\text{D}}} + 1/2*{\text{d}}_{{\text{M}}} }}{{{\text{d}}_{{\text{D}}} + {\text{d}}_{{{\text{ND}}}} {\text{ + d}}{}_{{\text{M}}}}}$, where *d*_*D*_, *d*_*ND*_ and *d*_*M*_ denote durations of deprived eye exclusive percept, non-deprived eye exclusive percept and mixed percept (i.e., the summed duration of superimposition and piecemeal percepts), respectively. We added half of the mixed percept duration to both the dominant and non-dominant eye percept durations to reflect the contribution of each eye in both exclusive and mixed percepts. Average numbers of alternations per second (i.e., alternation rates) were also calculated. The duration of mixed percepts and alternation rate were secondary outcome measures.

In the letter-polarity test (Fig. 1), participants viewed two pairs of dichoptic letters with different contrasts. The contrasts of letters on each side added up to zero, with the brighter-than-background letters presented randomly in the top or bottom position. The two bright letters on both sides were shown on different rows. Thus, when participants viewed the stimuli, there was always one darker and one brighter component for each top and bottom letter. On each trial, participants reported whether the upper or lower letter was brighter. Different letter contrasts were presented using the method of constant stimuli to measure the “balance point” at which both letters were equally likely to be perceived as brighter. The interocular contrast difference varied from 0 to 0.4 (corresponding letter contrasts were 0.3–0.7, in steps of 0.05) over 180 trials (9 contrasts × 20 repetitions). Full details of the letter contrast manipulations are provided by Bossi et al.^23,24^. The point of subjective equality (PSE), i.e., the “balance point” or ocular dominance value, was calculated after curve fitting using a Logistic function. In both tests, an ocular dominance value of 0.5 indicated equal dominance of both eyes, and a value larger than 0.5 indicated deprived eye dominance.

### Transcranial direct current stimulation (tDCS)

In both Experiment 1 (2-h patching) and Experiment 2 (30-min patching), tDCS was delivered using a battery-driven stimulator (NeuroConn DC-Stimulator Plus). Towards the end of Experiment 2, we switched to a different stimulator (NeuroConn DC-Stimulator MC) while using the same stimulation protocol. This switch only affected three visits. Two 5 × 7 electrodes were used, soaked in saline sponges. The target electrode was placed at Oz, and the reference electrode was placed at Cz, as defined by the International 10/20 Electrode Positioning System. Direct current at 2 mA was delivered for 20 min with 20-s ramp up and ramp down periods. During sham stimulation, the current ramped up and then immediately down. Anodal, cathodal and sham stimulation sessions occurred on different days with an interval of at least 48 h. The stimulation sequence was counterbalanced. Participants were not informed of the type of stimulation being delivered each day.

### Procedures

Each participant had three visits. On each visit, baseline ocular dominance was measured using both the grating rivalry test and the letter-polarity test. The sequence of these two tests was counterbalanced across participants. The Miles eye dominance test was also performed. Participants extended their arms before them, formed a triangular aperture with their hands and viewed a distant object through the aperture. The dominant eye retained the image of the object when each eye viewed monocularly. This sighting test allowed a dominant eye to be determined if dominance measures were not consistent between the grating rivalry and letter-polarity test. The dominant eye was subsequently deprived with a translucent eye patch (monocular deprivation, MD) for 2 h (Experiment 1) or 30 min (Experiment 2) (Fig. 1). Participants were instructed to keep both eyes open and watched a common sequence of comedy videos during this time. tDCS (anodal, cathodal, or sham stimulation) was delivered during the final 20 min of MD. Both computerized ocular dominance tests were repeated immediately after patching. Additionally, in Experiment 1, the letter-polarity test was repeated after 90 min of MD (i.e., 10 min before tDCS started). We chose this test during patching to minimize disruption to the MD effect as it was shorter than the grating rivalry test (letter test mean 3.34 ± SD 1.47 min versus grating test 6 min). A questionnaire was provided at the end of each session to document any possible side effects of brain stimulation.

### Data analysis

Data were analyzed using JASP (Version 0.16.2.0). Normality of the data was examined using Shapiro–Wilk tests. For normally distributed data, a two-way repeated measures ANOVA was used to assess the effect of Condition (a-tDCS, c-tDCS and Sham) and Time (Pre, PreStim and Post). Effect sizes (omega squared, ω^2^) were reported for these analyses. For nonparametric data, Friedman tests were used in place of repeated measures ANOVAs. Effect sizes were illustrated using Kendall’s *W*. Pairwise comparisons (independent samples *t* tests or Mann–Whitney *U* tests) were used to compare outcomes between the two experiments.

## Results

### Experiment 1: 120-min MD

Twenty participants were screened. Two were excluded due to vision not reaching 6/6 in one eye and one was excluded due to unstable fusion. Therefore, a total of 17 participants (age: 21–28, mean 24.41 years, 10 females) completed the experiment.

#### Primary outcome: deprived eye dominance

**Figure 2:**
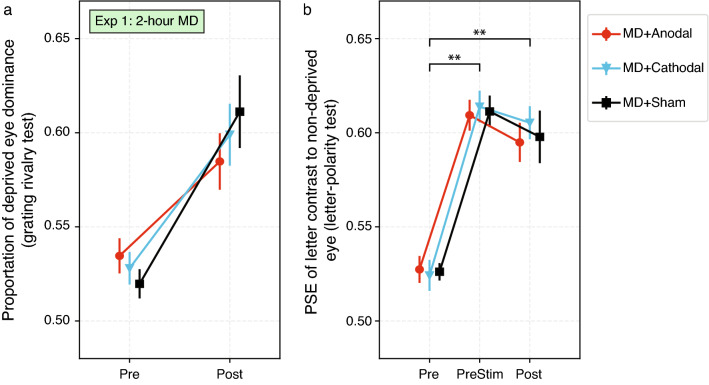
Deprived eye dominance at different time points for each tDCS condition in Experiment 1. (**a**) For the grating rivalry test, the proportion of deprived eye dominance is illustrated. Ocular dominance was measured at baseline (Pre) and immediately after MD (Post). (**b**) For the letter-polarity test, deprived eye dominance was indicated by the non-deprived eye letter contrast at the point of subjective equality (PSE). Ocular dominance was measured at baseline (Pre), 90 min after MD started (PreStim), and immediately after MD (Post). Ocular dominance > 0.5 indicates deprived eye dominance. Error bars represent standard errors of the mean. **Post hoc *t* tests *p* < 0.001.

Changes in deprived eye dominance are shown in Fig. 2. For the grating rivalry test (Fig. 2a), deprived eye dominance significantly increased after MD (Time: χ^2^ = 22.4, *p* < 0.001, *W* = 0.563). There was no significant effect of Condition (χ^2^ = 0.40, *p* = 0.819, *W* = 0.019). For the letter-polarity test (Fig. 2b), deprived eye dominance significantly increased after MD (Time: *F*_2,30_ = 152.1, *p* < 0.001, ω^2^ = 0.592). Post hoc tests showed that eye dominance increased significantly at the pre-stim timepoint (90 min of MD immediately before tDCS), then remained stable after tDCS (Pre vs PreStim *p* < 0.001, Pre vs Post *p* < 0.001, PreStim vs Post *p* = 0.157). There was no significant effect of Condition (*F*_2,30_ = 0.181, *p* = 0.835, ω^2^ < 0.001) and no interaction (*F*_2,30_ = 0.238, *p* = 0.790, ω^2^ < 0.001).

#### Secondary outcome: mixed percepts and alternation rate

In the grating rivalry test, participants used two buttons to indicate their superimposition and piecemeal percepts. The durations of these two percepts were summed to give the overall duration of mixed percepts. Supplementary Fig. S1 shows the changes in these percepts. Only overall mixed percept data were normally distributed. Superimposition, piecemeal and overall mixed percept durations did not change significantly after MD (superimposition: χ^2^ = 0.579, *p* = 0.447, *W* = 0.026; piecemeal: χ^2^ = 1.386, *p* = 0.239, *W* = 0.009; overall mixed: *F*_1,15_ = 3.589, *p* = 0.078, ω^2^ = 0.009). In addition, tDCS did not modulate any of these percepts (superimposition: χ^2^ = 1.557, *p* = 0.459, *W* = 0.025; piecemeal: χ^2^ = 1.933, *p* = 0.380, *W* = 0.023; overall mixed: *F*_2,30_ = 0.038, *p* = 0.963, ω^2^ < 0.001). No interaction was found between Time and Condition for overall mixed percept (*F*_1.4,21.5_ = 0.125, *p* = 0.815, ω^2^ < 0.001).

Supplementary Fig. S2 shows changes in alternation rate as measured by the grating rivalry test. Alternation rate did not change significantly after MD (Time: *F*_1,15_ = 1.994, *p* = 0.178, ω^2^ = 0.004). tDCS had no effect on alternation rate (Condition: *F*_2,30_ = 3.270, *p* = 0.052, ω^2^ = 0.020). There was no interaction between Time and Condition (*F*_2,30_ = 1.719, *p* = 0.196, ω^2^ = 0.002).

### Experiment 2: 30-min MD

19 healthy adults were screened. Four were excluded due to vision not reaching 6/6 in one eye, and one due to stereoacuity not reaching 40 arcseconds. Three participants were excluded due to ocular dominance > 0.7. One participant withdrew due to “itchiness” following tDCS, though rated as mild at the end of their visit, and one participant withdrew due to personal reasons. Therefore, a total of 9 participants (age: 20–28, mean 23.44 years, 8 females) completed the experiment.

#### Primary outcome: deprived eye dominance

**Figure 3:**
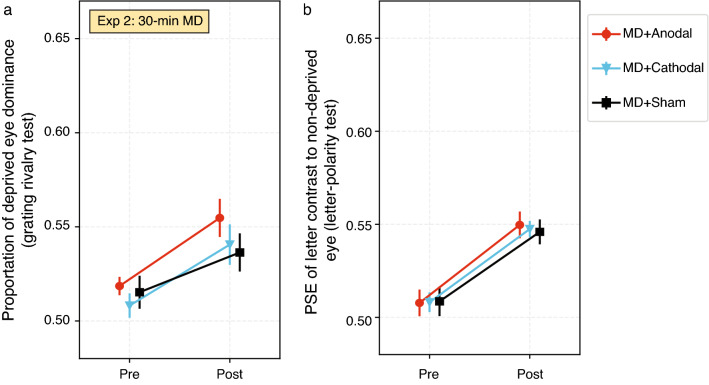
Deprived eye dominance data at different time points under each condition in Experiment 2. (**a**) Grating rivalry test. (**b**) Letter-polarity test. Ocular dominance was measured at baseline (Pre) and immediately after MD (Post). Error bars represent standard errors of the mean. Note that these plots share the same y-axis scale as Fig. 2 to facilitate comparison.

Changes in deprived eye dominance are shown in Fig. 3. For the grating rivalry test (Fig. 3a), deprived eye dominance significantly increased after MD (Time: *F*_1,8_ = 14.37, *p* = 0.005, ω^2^ = 0.307). There was no significant effect of Condition (*F*_2,16_ = 2.012, *p* = 0.166, ω^2^ = 0.031) and no interaction (*F*_2,16_ = 0.526, *p* = 0.601, ω^2^ < 0.001). For the letter-polarity test (Fig. 3b), deprived eye dominance significantly changed after MD (Time: *F*_1,8_ = 95.22, *p* < 0.001, ω^2^ = 0.564). There was no significant effect of Condition (*F*_2,16_ = 0.034, *p* = 0.967, ω^2^ < 0.001) and no interaction (*F*_2,16_ = 0.215, *p* = 0.809, ω^2^ < 0.001).

#### Secondary outcome: mixed percepts and alternation rate

Supplementary Fig. S3 shows the changes in the durations of superimposition, piecemeal and overall mixed percepts. As in Experiment 1, none of these percepts changed significantly after MD (superimposition: χ^2^ = 1.146, *p* = 0.284, *W* = 0.034; piecemeal: *F*_1,8_ = 1.939, *p* = 0.201, ω^2^ = 0.004; overall mixed: *F*_1,8_ = 4.107, *p* = 0.077, ω^2^ = 0.020). In addition, tDCS did not modulate any of these percepts (superimposition: χ^2^ = 1.244, *p* = 0.537, *W* = 0.040; piecemeal: *F*_2,16_ = 0.554, *p* = 0.585, ω^2^ < 0.001; overall mixed: *F*_2,16_ = 0.704, *p* = 0.509, ω^2^ < 0.001). No interaction was found between Time and Condition for piecemeal (*F*_2,16_ = 0.397, *p* = 0.679, ω^2^ < 0.001) or for overall mixed percept (*F*_2,16_ = 0.480, *p* = 0.628, ω^2^ < 0.001).

Supplementary Fig. S4 shows changes in alternation rate as measured by the grating rivalry test. Alternation rate did not change significantly after MD (Time: *F*_1,8_ = 0.562, *p* = 0.475, ω^2^ < 0.001). tDCS had no effect on alternation rate (Condition: *F*_2,16_ = 0.869, *p* = 0.438, ω^2^ < 0.001). There was no interaction between Time and Condition (*F*_2,16_ = 0.718, *p* = 0.503, ω^2^ < 0.001).

### Comparison between Experiments 1 and 2: effect of MD duration on ocular dominance plasticity

Because we did not observe any effect of tDCS, we calculated a mean ocular dominance change for each participant across the three tDCS conditions and compared these means between Experiment 1 (120-min MD) and Experiment 2 (30-min MD). For the grating rivalry test, the ocular dominance changes in Experiment 1 (Fig. 2a, mean 0.071 ± SE 0.014) were significantly larger than those changes in Experiment 2 (Fig. 3a, 0.030 ± 0.008) (*U* = 117.0, *p* = 0.029). For the letter-polarity test, ocular dominance changes were also significantly larger in Experiment 1 (Fig. 2b, 0.073 ± 0.006) than in Experiment 2 (Fig. 3b, 0.040 ± 0.004) (*t* = 4.113, *p* < 0.001).

## Discussion

We first tested whether anodal, cathodal or sham tDCS had an effect on short-term ocular dominance plasticity induced by 2 h of MD. As anodal tDCS has been reported to reduce GABA inhibition^5–7,9^, we hypothesized that the reduced inhibition would augment ocular dominance changes following MD. While the MD effect was significant, we did not observe an effect of a-tDCS. In a second experiment, we investigated whether there was a ceiling effect for ocular dominance plasticity induced by 2 h of MD by reducing the MD duration to 30 min. This second experiment demonstrated that the MD effect was significantly smaller for 30 min of MD. However, again we did not observe any effect of a-tDCS. In both experiments we did not observe any significant effects of a-tDCS on binocular rivalry mixed percepts or alternation rate.

Using a similar experimental design (where ocular dominance plasticity was induced by MD), we previously observed no effect of transcranial random noise stimulation (tRNS) on ocular dominance plasticity^22^. tRNS may augment subthreshold signals via stochastic resonance^25^ and by reducing GABAergic inhibition in the stimulated cortex^26,27^, thereby leading to improved resolution of visual stimuli. We concluded that there were at least two possible explanations for our null results, either that tRNS does not modulate the ocular dominance changes induced by MD or that 2 h of MD produces the maximum possible amount of ocular dominance plasticity (a ceiling effect). The neuromodulatory effects of tDCS may differ from those of tRNS. The induction of a constant electric current influences the activity of sodium and calcium channels on neuron membranes^4,5^. Specifically, the anodal electrode increases the probability of channels opening on the soma (i.e., cell body) membrane of stimulated neurons, resulting in an influx of sodium and calcium ions and a higher resting membrane potential. Neurons are then more likely to fire an action potential when presented with a visual stimulus. Modulation of the GABAergic system may also be an important mechanism for enhanced neuroplasticity^28^. Taken together, our two studies suggest that even with potentially differing mechanisms, stimulation of the visual cortex using either tRNS or tDCS does not alter ocular dominance plasticity resulting from MD.

Although many studies have reported a-tDCS effects on visual cortex function and plasticity^10–16^, our study is not the first to observe no effect. For instance, while Ding et al.^16^ and Frase et al.^10^ demonstrated a modulation of VEP amplitude using a-tDCS, other studies^29,30^ did not observe such an effect. Abuleil et al.^31^ observed that tDCS did not modulate binocular rivalry dynamics, while a type of repetitive transcranial magnetic stimulation, namely continuous theta burst stimulation (cTBS), had an effect. Lau et al.^29^ pointed out that tDCS effects on vision tasks can differ depending on whether tasks are performed during (“online”) or after (“offline”) tDCS. Our study used an offline design (i.e., outcome measures occurring after tDCS), as did most studies mentioned above that observed modulatory effects of tDCS. Prior tDCS studies have investigated various types of visual cortex plasticity, including Hebbian plasticity (e.g., perceptual learning) and homeostatic plasticity (e.g., ocular dominance plasticity). The distinct mechanisms underlying these different types of plasticity^32^ may explain why tDCS had effects on some types of plasticity but not on ocular dominance plasticity.

Our hypothesis for an a-tDCS effect on ocular dominance plasticity was based on studies of the human motor cortex that showed reduced GABA inhibition following stimulation^5–7^. However, it remains an open question whether a-tDCS exerts the same effect on GABA concentration when applied to the visual cortex. In cats, a-tDCS was found to increase the neuronal response to a light stimulus whereas c-tDCS reduced the response^33^. It has also been shown that a-tDCS reduces GABA concentration while c-tDCS reduces glutamate concentration in the cat visual cortex^8^. In humans, visual cortex a-tDCS increases gamma oscillations measured using MEG, an indirect measure of reduced GABA concentration^34^. However, other studies using indirect behavioural measures linked to visual cortex GABA concentration have observed no effect of a-tDCS^31^. A differential effect of a-tDCS on motor versus visual cortex GABA concentration might explain the null effect of a-tDCS in our study.

Various factors can influence the effect of tDCS including the polarity of the electrode placed above the targeted cortical area (anode vs cathode), the relative location of the stimulating and reference electrodes, electrode size and the intensity and duration of the stimulating current^35^. Individual differences in cortical and cranial anatomy may also influence tDCS effects^36^. The tDCS parameters used in this experiment (i.e., the stimulating electrode positioned at Oz and the reference electrode at Cz, 5 × 7 cm^2^ electrode size, 2 mA current and 20-min stimulation) have been used in previous studies, some of which reported stimulation effects^12–16^ while others did not^29–31^. However, a wide variety of alternative parameters could have been used and we did not attempt to model and account for anatomical differences between subjects. Therefore, our null results should be interpreted within the context of the specific tDCS parameters used and the age and sex characteristics of our sample.

The timing of tDCS in relation to MD may also matter. Some studies show that tDCS enhances motor training more when applied concurrently than applied before training^37,38^, while other studies report that it is more beneficial to apply tDCS prior to training than concurrently with training^39–41^. To our knowledge, the effect of tDCS timing has not yet been investigated in the visual cortex. While we observed that a-tDCS applied at the end of MD did not modulate ocular dominance plasticity, it is possible that a-tDCS delivered prior to or at the beginning of MD could influence ocular dominance changes.

Based on our null findings from both the tRNS study^22^ and Experiment 1 in this tDCS study, we hypothesized that 2 h of MD may induce a ceiling effect for ocular dominance plasticity. Our second experiment showed that shorter MD does result in a smaller ocular dominance shift whereby the ocular dominance change after 2 h of MD was approximately onefold greater than the change after 30 min of MD. Nevertheless, we still did not observe an effect of a-tDCS applied at the end of 30 min of MD. This makes an explanation for our null results based on a ceiling effect less likely. However, we cannot rule out yet that an even shorter duration of MD might reveal an a-tDCS effect.

We did not observe an effect of a-tDCS on ocular dominance plasticity in adults with normal vision. However, the mechanisms underlying homeostatic plasticity may be different from those that underpin plasticity associated with vision rehabilitation. Whether tDCS enhances ocular dominance plasticity for patients with binocular visual impairments such as amblyopia is currently unknown.

## Conclusions

This study investigated the effects of tDCS and the duration of MD on ocular dominance plasticity. Shorter MD induced smaller ocular dominance changes. With both longer MD and shorter MD, however, we did not observe any difference between anodal, cathodal or sham tDCS conditions. It remains possible that tDCS applied prior to or at the beginning of MD could influence ocular dominance changes. Future studies could investigate the effect of tDCS timing when applied to the visual cortex, and whether tDCS influences ocular dominance plasticity in patients with visual impairment.

## Supplementary Information

Supplementary Information.

## Data Availability

Raw data for the current study are available in the Figshare repository, https://figshare.com/articles/dataset/tDCS_MD_data_2022_/21928398.
