# Supplementary material for: Short-term ocular dominance plasticity is not modulated by visual cortex tDCS but increases with length of monocular deprivation
Source: Sci Rep. 2023 Apr 24;13:6666. doi: 10.1038/s41598-023-33823-7 (PMC10126033; doi:10.1038/s41598-023-33823-7)
Supplement: Supplementary file 1 — Supplementary Information. [file 41598_2023_33823_MOESM1_ESM.pdf]

Short-term ocular dominance plasticity is not modulated by visual cortex tDCS but increases with length of monocular deprivation

Authors: Xiaoxin Chen, William Bobier, Benjamin Thompson

Supplementary Figures

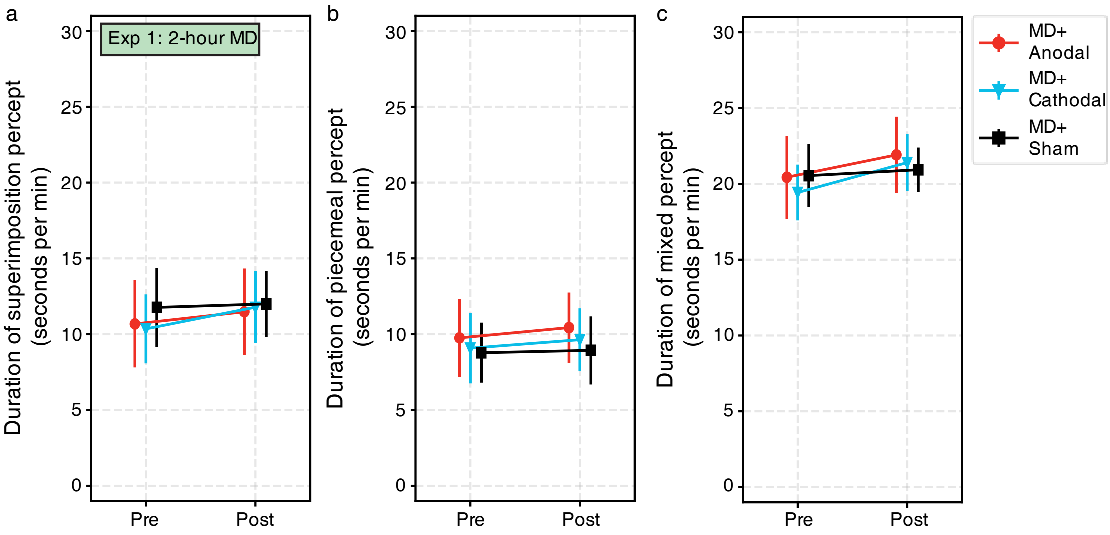

Figure S1. Duration of superimposition (a), piecemeal (b) and overall mixed (c) percepts at Pre and Post MD for each tDCS condition in Experiment 1. The durations were averaged across six trials of the grating rivalry test. Error bars represent standard errors of the mean.

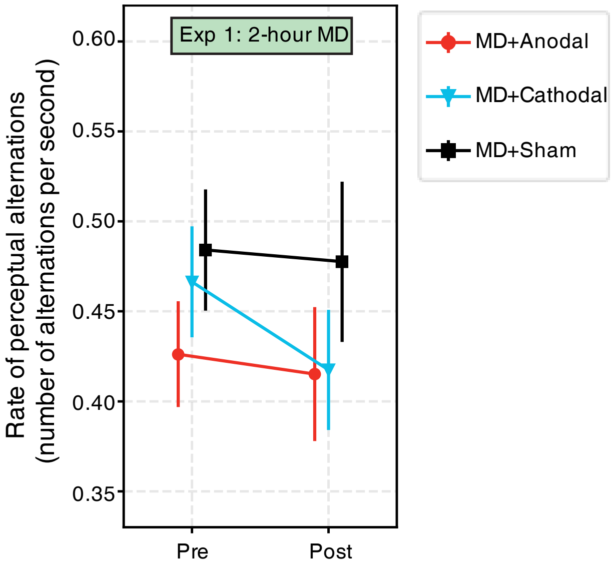

Figure S2. Rate of alternations at Pre and Post MD for each tDCS condition in Experiment 1. Alternation rate was averaged across six trials of a grating rivalry test. Error bars represent standard errors of the mean.

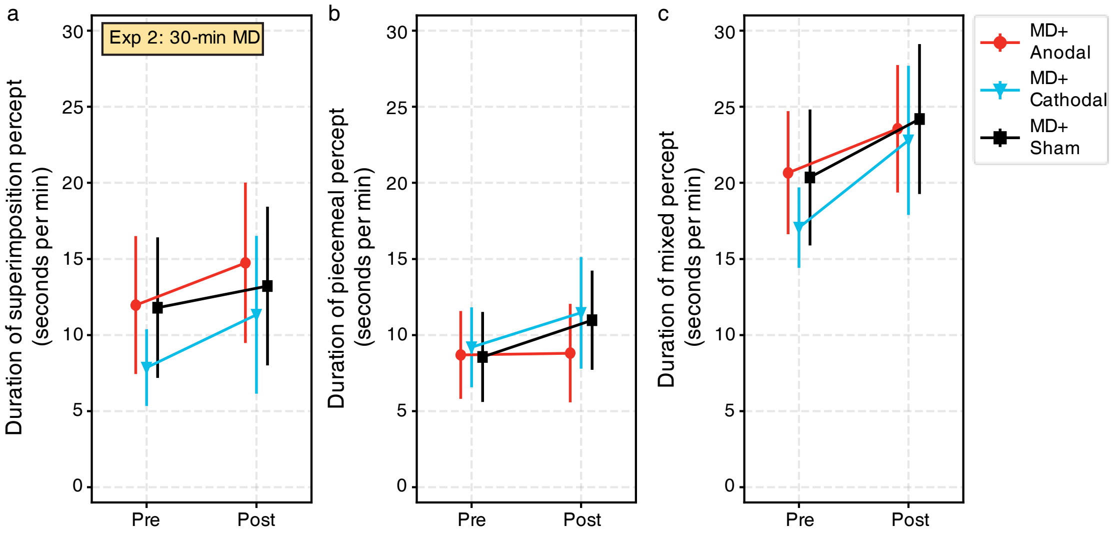

Figure S3. Duration of superimposition (a), piecemeal (b) and overall mixed (c) percepts at Pre and Post under each condition in Experiment 2. The durations were averaged across six trials of a grating rivalry test. Error bars represent standard errors of the mean.

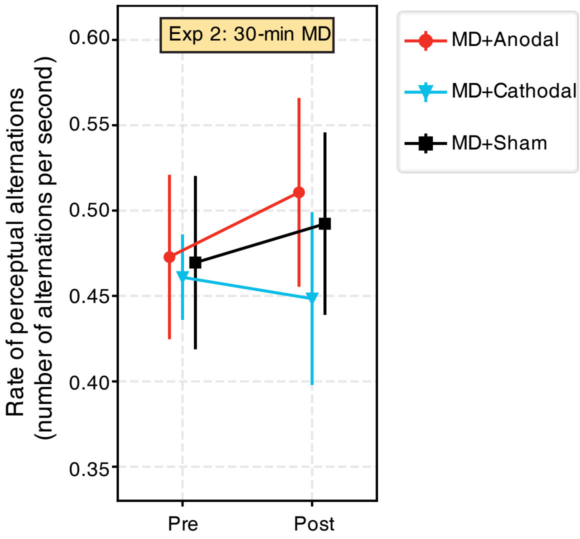

Figure S4. Rate of alternations at Pre and Post MD for each condition in Experiment 2. Alternation rate was averaged across six trials of a grating rivalry

test. Error bars represent standard errors of the mean.

Supplementary Table S1 Reports of adverse effects of tDCS.

**Anodal**	**Cathodal**	**Sham**
Skin redness (21/26)	Skin redness (21/26)	Skin redness (12/26)
Tingling (3/26)	Tingling (7/26)	Tingling (2/26)
Itching (5/26)	Itching (4/26)	Itching (1/26)
Headache (4/26)	Headache (1/26)	Headache (2/26)
Scalp pain (1/26)	Scalp pain (4/26)	Scalp pain (0/26)
Burning sensation (0/26)	Burning sensation (2/26)	Burning sensation (0/26)
Neck pain (1/26)	Neck pain (0/26)	Neck pain (0/26)
Sleepiness (4/26)	Sleepiness (4/26)	Sleepiness (5/26)
Trouble concentrating (2/26)	Trouble concentrating (2/26)	Trouble concentrating (2/26)

All reports were rated as mild, except for a total of six moderate-level reports of "skin redness" (one anodal, one cathodal), "tingling" (one anodal), "sleepiness" (one anodal and one sham) and "trouble concentrating" (one sham).

The prevalent reports of mild skin redness in this study could be partially due to the tightness of bands holding the electrodes in place and manual pressing on the electrodes to improve conductivity, as corroborated by 12 reports in the sham condition.
